# [2,3,7,8,13,14,17,18-Octa­kis(ethyl­sulfan­yl)-5,10,15,20-porphyrazinato]zinc(II)

**DOI:** 10.1107/S1600536810028588

**Published:** 2010-07-24

**Authors:** Mehmet Akkurt, Naciye Yılmaz Coşkun, Fatma Aytan Kılıçaslan, Sabiha Manav Yalçın, Orhan Büyükgüngör, Ahmet Gül

**Affiliations:** a Department of Physics, Faculty of Arts and Sciences, Erciyes University, 38039 Kayseri, Turkey; b Department of Chemistry, Faculty of Arts and Sciences, Yıldız Technical University, 34210 Esenler, Istanbul, Turkey; c Department of Physics, Faculty of Arts and Sciences, Ondokuz Mayıs University, 55139 Samsun, Turkey; d Department of Chemistry, Technical University of Istanbul, 34469 Maslak, Ístanbul, Turkey

## Abstract

In the title compound, [Zn(C_32_H_40_N_8_S_8_)], the Zn^II^ ion is coordinated by four N atoms in a slightly distorted square-planar environment. In addition, there is a Zn⋯S contact involving a symmetry-related S atom which, when considered, forms a pseudo-square-pyramidal coordination with respect to the Zn^II^ ion. Three of the ethyl groups are disordered over two sites with occupancy ratios of 0.841 (10):0.159 (10), 0.802 (10):0.198 (10) and 0.457 (13):0.543 (13). Weak intra­molecular C—H⋯N and C—H⋯S inter­actions contribute to the stability of the mol­ecular conformation. Inter­molecular C—H⋯S contacts, weak C—H⋯π inter­actions and π–π stacking inter­actions [centroid–centriod distances = 3.832 (4) and 3.850 (5) Å] contribute to the stabilization of the crystal structure.

## Related literature

For the synthesis of the title complex, see: Ricciardi *et al.* (2000 ▶); Belviso *et al.* (2000 ▶). For the synthesis and characterization of porphyrazines and their metal complexes, see: Schramm & Hoffman (1980 ▶); Sakellariou *et al.* (2000 ▶); Ramirez *et al.* (2004 ▶). For the Zr(IV), Mn(III), Fe(III), Cu(II), Ni(II) and some lanthanide complexes of (ethyl­sulfan­yl) porphyrazines, see: Ricciardi *et al.*(1996*a* ▶,*b* ▶, 1998 ▶, 1999 ▶).
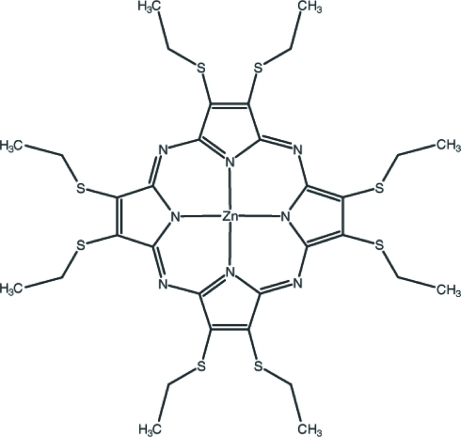


## Experimental

### 

#### Crystal data

[Zn(C_32_H_40_N_8_S_8_)]
*M* *_r_* = 858.67
Orthorhombic, 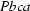

*a* = 8.7973 (1) Å
*b* = 27.2813 (3) Å
*c* = 32.0903 (6) Å
*V* = 7701.73 (19) Å^3^
*Z* = 8
Mo *K*α radiation
μ = 1.11 mm^−1^
*T* = 295 K
0.60 × 0.37 × 0.13 mm

#### Data collection

Stoe IPDS 2 diffractometer
Absorption correction: part of the refinement model (Δ*F*) (*XABS2*; Parkin *et al.*, 1995 ▶) *T*_min_ = 0.620, *T*_max_ = 0.866
7282 measured reflections
7282 independent reflections
5696 reflections with *I* > 2σ(*I*)

#### Refinement

*R*[*F* ^2^ > 2σ(*F* ^2^)] = 0.040
*wR*(*F* ^2^) = 0.097
*S* = 1.05
7282 reflections
466 parameters
16 restraints
H-atom parameters constrained
Δρ_max_ = 0.44 e Å^−3^
Δρ_min_ = −0.30 e Å^−3^

### 

Data collection: *X-AREA* (Stoe & Cie, 2002 ▶); cell refinement: *X-AREA*; data reduction: *X-RED32* (Stoe & Cie, 2002 ▶); program(s) used to solve structure: *SIR97* (Altomare *et al.*, 1999 ▶); program(s) used to refine structure: *SHELXL97* (Sheldrick, 2008 ▶); molecular graphics: *ORTEP-3* (Farrugia, 1997 ▶); software used to prepare material for publication: *WinGX* (Farrugia, 1999 ▶).

## Supplementary Material

Crystal structure: contains datablocks global, I. DOI: 10.1107/S1600536810028588/lh5086sup1.cif

Structure factors: contains datablocks I. DOI: 10.1107/S1600536810028588/lh5086Isup2.hkl

Additional supplementary materials:  crystallographic information; 3D view; checkCIF report

## Figures and Tables

**Table 1:** Selected bond lengths (Å)

Zn1—N1	2.004 (2)
Zn1—N3	1.994 (2)
Zn1—N5	2.004 (2)
Zn1—N7	1.994 (2)
Zn1—S5^i^	2.6364 (9)

Symmetry code: (i) 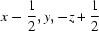.

**Table 2:** Hydrogen-bond geometry (Å, °) *Cg*1, *Cg*2 and *Cg*3 are the centroids of the N3/C9–C12, Zn1/N1/N7/N8/C1/C26 and Zn1/N3/N4/N5/C10/C17 rings, respectively.

*D*—H⋯*A*	*D*—H	H⋯*A*	*D*⋯*A*	*D*—H⋯*A*
C5*A*—H5*A*1⋯S1^ii^	0.97	2.86	3.764 (5)	155
C7—H7*A*⋯N2	0.97	2.50	3.014 (5)	113
C21—H21*A*⋯S4^i^	0.97	2.77	3.660 (4)	154
C23—H23*B*⋯S5	0.97	2.69	3.221 (4)	115
C31*B*—H31*D*⋯N8	0.97	2.53	3.089 (9)	116
C21—H21*B*⋯*Cg*3^iii^	0.97	2.86	3.536 (3)	128
C22—H22*A*⋯*Cg*1^iii^	0.96	2.90	3.699 (4)	142
C23—H23*B*⋯*Cg*2^iii^	0.97	2.81	3.641 (4)	144

Symmetry codes: (i) 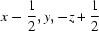; (ii) 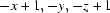; (iii) 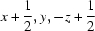.

**Fig. 1.**
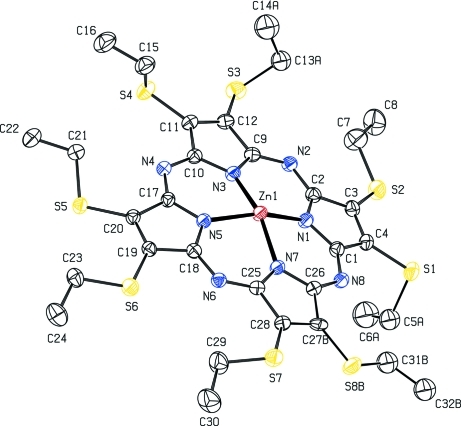
The title molecule with the atom numbering scheme. Displacement ellipsoids for non-H atoms are drawn at the 30% probability level. The minor occupied sites of the disordered atoms and H atoms have been omitted for clarity.

**Fig. 2.**
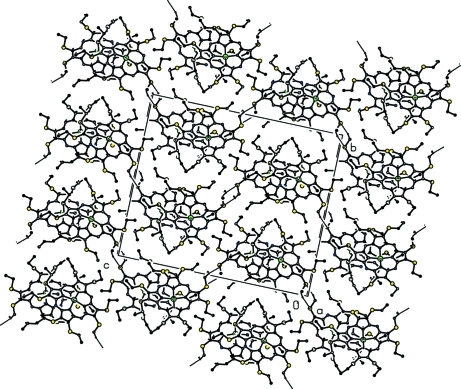
The crystal packing of the major component of (I) viewed down the a axis. All hydrogen atoms not involved in hydrogen bonding have been omitted for clarity.

## supplementary crystallographic information

### Comment

The synthesis and characterization of porphyrazines (tetrapyrrole macrocycles) and their metal complexes are a topic of growing interest (Schramm & Hoffman, 1980). They have high symmetry, planarity, thermal stability and electronic delocalization. So, these types of compounds have potential applications for interesting optical, electrical, medical and catalytic properties. One synthetic route to octakis-functionalized porphyrazine is the cyclization of the functionalized dicyano precursor in the presence of magnesium alkoxide (Sakellariou *et al.*, 2000; Ramirez *et al.*, 2004). Their properties can be easily modified by attachment of diverse peripheral substituents, heteroatoms or alternation of the cetral metal ion.

The Zr(IV), Mn(III), Fe(III), Cu(II), Ni(II) and some lantanides complexes of (ethylsulfanyl) porphyrazines have been investigated (Ricciardi *et al.*, 1996*a*,*b*; 1998; 1999). In this present work, we report the crystal structure of the title compound (I).

In the molecule of (I) shown in Fig. 1, the Zn—N bond distances range from from 1.994 (2) to 2.004 (2) Å. The intermolecular Zn—S distance [2.6364 (9) Å] which leads to a pseudo-square-pyramidal coordination around the Zn^II^ ion, is shorter than the Co—S distances [alternatively 2.789 (5) and 2.842 (5) Å] in ((ethylsulfanyl)porphyrazinato)cobalt(II) (Ricciardi *et al.*, 1999).

The molecular conformation of (I) is stabilized by weak intramolecular C—H···N and C—H···S interactions. In the crystal structure, intermolecular C—H···S contacts (Fig. 2), weak C—H···π interactions and π-π stacking interactions [*Cg*3···*Cg*4*^ii^* = 3.832 (4) Å and *Cg*3···*Cg*5*^ii ^*= 3.850 (5) Å; symmetry code: (ii) 1/2 + *x*, *y*, 1/2 - *z*; *Cg*3, *Cg*4 and *Cg*5 are the centroids of the N5/C17–C20, N7/C25/C26/C27B/C28 and N7/C25/C26/C27A/C28 rings, respectively] contribute to the stabilization of the crystal structure.

### Experimental

All starting materials and reagents used were of standard analytical grade from Merck, Fluka and Aldrich. The title complex were synthesized according to literature (Ricciardi *et al.*, 2000; Belviso *et al.*, 2000).

2,3,7,8,13,17,18-Octakis(ethylsulfanyl)-5,10,15,20-porphyrazine (0.1 g, 0.12 mmol) was dissolved in CHCl_3_ and added to the solution of Zn(CH_3_COO)_2_.4H_2_O (0.306 g, 1.2 mmol) in EtOH. The mixture was refluxed under argon for 1 h. After cooling to room temperature, insoluble excess Zn(CH_3_COO)_2_ was separeted by filtering. The filtrate was evaporated and the resulting deep blue solid was purified by chromatography on silica gel using CHCl_3_. For single-crystal, compound (I) has been crystalized at CHCl_3_/ MeOH (1/9).

### Refinement

H atoms were positioned geometrically with C—H = 0.96 and 0.97 Å and refined using a riding model with *U*_iso_(H) = 1.2 or 1.5*U*_eq_(C). The three ethyl groups of the title molecule are disordered over two sites in the 0.841 (10):0.159 (10) for C5A/C6A:C5B/C6B, 0.802 (10):0.198 (10) for C13A/C14A:C13B/C14B and 0.457 (13):0.543 (13) for C32A/C33A:C32B/C33B ratios and they were refined isotropicaly for a stable refinement. The S (S8A:S8B) atom with the third ethyl group (C32A/C33A:C32B/C33B) and the C atom (C27A:C27B) of the pyrrole ring attached to are also disorder over two sites in a 0.457 (13):0.543 (13) ratio. In the disorder segments of (I), the *DFIX* instructions were used to constrain the bond lengths to reasonable values.

### Crystal data

**Table:**

[Zn(C_32_H_40_N_8_S_8_)]	*F*(000) = 3568
*M**_r_* = 858.67	*D*_x_ = 1.481 Mg m^−^^3^
Orthorhombic, *P**b**c**a*	Mo *K*α radiation, λ = 0.71073 Å
Hall symbol: -P 2ac 2ab	Cell parameters from 86381 reflections
*a* = 8.7973 (1) Å	θ = 1.3–26.2°
*b* = 27.2813 (3) Å	µ = 1.11 mm^−^^1^
*c* = 32.0903 (6) Å	*T* = 295 K
*V* = 7701.73 (19) Å^3^	Prism, black
*Z* = 8	0.60 × 0.37 × 0.13 mm

### Data collection

**Table:**

Stoe IPDS 2 diffractometer	7282 independent reflections
Radiation source: sealed X-ray tube, 12 x 0.4 mm long-fine focus	5696 reflections with *I* > 2σ(*I*)
plane graphite	*R*_int_ = 0.0000
Detector resolution: 6.67 pixels mm^-1^	θ_max_ = 25.7°, θ_min_ = 1.3°
ω scans	*h* = 0→10
Absorption correction: part of the refinement model (Δ*F*) (*XABS2*; Parkin *et al.*, 1995)	*k* = 0→33
*T*_min_ = 0.620, *T*_max_ = 0.866	*l* = 0→38
7282 measured reflections	

### Refinement

**Table:**

Refinement on *F*^2^	Primary atom site location: structure-invariant direct methods
Least-squares matrix: full	Secondary atom site location: difference Fourier map
*R*[*F*^2^ > 2σ(*F*^2^)] = 0.040	Hydrogen site location: inferred from neighbouring sites
*wR*(*F*^2^) = 0.097	H-atom parameters constrained
*S* = 1.05	*w* = 1/[σ^2^(*F*_o_^2^) + (0.0526*P*)^2^ + 1.401*P*] where *P* = (*F*_o_^2^ + 2*F*_c_^2^)/3
7282 reflections	(Δ/σ)_max_ = 0.001
466 parameters	Δρ_max_ = 0.44 e Å^−^^3^
16 restraints	Δρ_min_ = −0.30 e Å^−^^3^

### Special details

**Table:**

Experimental. (XABS2; Parkin *et al.*, 1995; Cubic fit to sin(theta)/lambda - 24 parameters)
Geometry. Bond distances, angles *etc*. have been calculated using the rounded fractional coordinates. All su's are estimated from the variances of the (full) variance-covariance matrix. The cell e.s.d.'s are taken into account in the estimation of distances, angles and torsion angles
Refinement. Refinement on *F*^2^ for ALL reflections except those flagged by the user for potential systematic errors. Weighted *R*-factors *wR* and all goodnesses of fit *S* are based on *F*^2^, conventional *R*-factors *R* are based on *F*, with *F* set to zero for negative *F*^2^. The observed criterion of *F*^2^ > σ(*F*^2^) is used only for calculating -*R*-factor-obs *etc*. and is not relevant to the choice of reflections for refinement. *R*-factors based on *F*^2^ are statistically about twice as large as those based on *F*, and *R*-factors based on ALL data will be even larger.

### Fractional atomic coordinates and isotropic or equivalent isotropic displacement parameters (Å^2^)

**Table:**

	*x*	*y*	*z*	*U*_iso_*/*U*_eq_	Occ. (<1)
Zn1	0.62155 (4)	0.18388 (1)	0.32349 (1)	0.0401 (1)	
S1	0.56218 (12)	0.06894 (3)	0.47611 (3)	0.0617 (3)	
S2	0.79562 (12)	0.16274 (3)	0.50015 (2)	0.0623 (3)	
S3	1.05348 (11)	0.31228 (3)	0.40786 (3)	0.0598 (3)	
S4	1.05456 (10)	0.34318 (3)	0.30195 (3)	0.0579 (3)	
S5	0.88817 (9)	0.24250 (3)	0.16339 (2)	0.0441 (2)	
S6	0.60435 (10)	0.14544 (3)	0.14841 (2)	0.0541 (3)	
S7	0.27372 (11)	0.03081 (3)	0.23777 (3)	0.0579 (3)	
S8B	0.3114 (11)	−0.0068 (2)	0.33482 (16)	0.0732 (15)	0.543 (13)
N1	0.6525 (3)	0.15882 (8)	0.38158 (7)	0.0413 (8)	
N2	0.8203 (3)	0.22106 (8)	0.40843 (7)	0.0410 (8)	
N3	0.7915 (3)	0.23069 (8)	0.33388 (7)	0.0389 (7)	
N4	0.8438 (3)	0.25778 (8)	0.26365 (7)	0.0385 (7)	
N5	0.6573 (3)	0.19305 (8)	0.26230 (7)	0.0381 (7)	
N6	0.5007 (3)	0.12757 (8)	0.23537 (7)	0.0400 (7)	
N7	0.5143 (3)	0.12142 (8)	0.31024 (7)	0.0406 (7)	
N8	0.4965 (3)	0.08565 (8)	0.37884 (7)	0.0433 (8)	
C1	0.5853 (3)	0.11832 (10)	0.39820 (9)	0.0418 (9)	
C2	0.7336 (3)	0.18153 (10)	0.41229 (9)	0.0421 (9)	
C3	0.7156 (3)	0.15368 (11)	0.45124 (9)	0.0446 (9)	
C4	0.6246 (4)	0.11465 (11)	0.44202 (8)	0.0451 (9)	
C5A	0.6702 (6)	0.01709 (18)	0.45794 (17)	0.0751 (18)*	0.841 (10)
C6A	0.8372 (8)	0.0233 (3)	0.4623 (3)	0.120 (3)*	0.841 (10)
C7	0.8662 (6)	0.22413 (16)	0.50147 (13)	0.0883 (18)	
C8	0.9413 (6)	0.2340 (2)	0.54207 (13)	0.101 (2)	
C9	0.8469 (3)	0.24323 (10)	0.37194 (9)	0.0407 (9)	
C10	0.8562 (3)	0.26070 (10)	0.30526 (9)	0.0389 (8)	
C11	0.9524 (3)	0.29630 (10)	0.32636 (9)	0.0424 (9)	
C12	0.9476 (3)	0.28539 (10)	0.36767 (9)	0.0438 (9)	
C13A	0.9045 (6)	0.33738 (18)	0.44078 (16)	0.0690 (17)*	0.802 (10)
C14A	0.8356 (9)	0.3835 (3)	0.4200 (3)	0.107 (2)*	0.802 (10)
C15	0.9011 (4)	0.37875 (13)	0.27885 (13)	0.0690 (14)	
C16	0.9640 (6)	0.41584 (14)	0.24931 (16)	0.092 (2)	
C17	0.7582 (3)	0.22486 (10)	0.24441 (8)	0.0372 (8)	
C18	0.5973 (3)	0.16502 (10)	0.23179 (8)	0.0387 (8)	
C19	0.6603 (3)	0.17977 (10)	0.19120 (8)	0.0384 (8)	
C20	0.7609 (3)	0.21698 (10)	0.19924 (8)	0.0385 (8)	
C21	0.9468 (4)	0.30188 (11)	0.18359 (11)	0.0552 (11)	
C22	1.0154 (5)	0.33019 (14)	0.14801 (12)	0.0753 (16)	
C23	0.6966 (4)	0.17078 (13)	0.10339 (10)	0.0573 (11)	
C24	0.6649 (5)	0.13667 (17)	0.06641 (12)	0.0817 (16)	
C25	0.4650 (3)	0.10740 (10)	0.27176 (9)	0.0394 (9)	
C26	0.4646 (3)	0.08733 (10)	0.33842 (9)	0.0403 (9)	
C27B	0.364 (3)	0.0494 (5)	0.3159 (4)	0.041 (4)	0.543 (13)
C28	0.3731 (3)	0.06272 (10)	0.27547 (9)	0.0416 (9)	
C29	0.3727 (5)	0.03955 (17)	0.18997 (12)	0.0837 (16)	
C30	0.3176 (6)	0.00225 (19)	0.15882 (14)	0.0970 (19)	
C31B	0.3468 (12)	−0.0166 (3)	0.3838 (2)	0.080 (3)*	0.543 (13)
C32B	0.2550 (14)	−0.0561 (4)	0.4038 (3)	0.085 (3)*	0.543 (13)
C31A	0.2574 (14)	0.0014 (4)	0.3878 (3)	0.076 (3)*	0.457 (13)
C32A	0.1888 (19)	−0.0470 (5)	0.4029 (4)	0.103 (4)*	0.457 (13)
C13B	0.960 (3)	0.3675 (9)	0.4188 (14)	0.174 (18)*	0.198 (10)
C5B	0.721 (3)	0.0346 (12)	0.4914 (8)	0.112 (14)*	0.159 (10)
C6B	0.815 (4)	0.0050 (19)	0.4767 (16)	0.145 (19)*	0.159 (10)
S8A	0.2570 (7)	0.0047 (3)	0.3375 (2)	0.0643 (14)	0.457 (13)
C14B	0.804 (2)	0.3711 (8)	0.4367 (7)	0.062 (6)*	0.198 (10)
C27A	0.386 (3)	0.0512 (7)	0.3183 (4)	0.044 (5)	0.457 (13)
H6A1	0.88780	−0.00550	0.45220	0.1800*	0.841 (10)
H8B	0.99020	0.26540	0.54110	0.1520*	
H6A2	0.86220	0.02840	0.49110	0.1800*	0.841 (10)
H6A3	0.86940	0.05120	0.44630	0.1800*	0.841 (10)
H7A	0.93860	0.22890	0.47900	0.1060*	
H7B	0.78290	0.24690	0.49750	0.1060*	
H8A	0.86650	0.23390	0.56380	0.1520*	
H5A1	0.63920	−0.01180	0.47340	0.0900*	0.841 (10)
H5A2	0.64620	0.01140	0.42880	0.0900*	0.841 (10)
H15B	0.84410	0.39520	0.30060	0.0820*	
H16A	1.03170	0.43740	0.26400	0.1380*	
H16B	0.88230	0.43450	0.23750	0.1380*	
H16C	1.01860	0.39940	0.22750	0.1380*	
H21A	0.85990	0.31930	0.19480	0.0660*	
H21B	1.02090	0.29770	0.20570	0.0660*	
H22A	1.09790	0.31180	0.13630	0.1130*	
H22B	1.05250	0.36110	0.15800	0.1130*	
H22C	0.93940	0.33570	0.12710	0.1130*	
H23A	0.65740	0.20330	0.09770	0.0690*	
H23B	0.80520	0.17330	0.10820	0.0690*	
H24A	0.71200	0.10550	0.07130	0.1230*	
H24B	0.70560	0.15090	0.04140	0.1230*	
H24C	0.55720	0.13230	0.06340	0.1230*	
H29A	0.35430	0.07240	0.17950	0.1000*	
H29B	0.48110	0.03570	0.19440	0.1000*	
H30A	0.34790	−0.02990	0.16760	0.1450*	
H30B	0.36100	0.00920	0.13200	0.1450*	
H30C	0.20880	0.00370	0.15700	0.1450*	
H31C	0.45350	−0.02490	0.38670	0.0950*	0.543 (13)
H31D	0.32970	0.01370	0.39900	0.0950*	0.543 (13)
H32D	0.15860	−0.04310	0.41240	0.1280*	0.543 (13)
H32E	0.30830	−0.06850	0.42770	0.1280*	0.543 (13)
H32F	0.23910	−0.08220	0.38420	0.1280*	0.543 (13)
H8C	1.01560	0.20900	0.54750	0.1520*	
H13A	0.82570	0.31300	0.44490	0.0830*	0.802 (10)
H13B	0.94620	0.34590	0.46780	0.0830*	0.802 (10)
H14A	0.91020	0.40920	0.41940	0.1610*	0.802 (10)
H14B	0.74860	0.39420	0.43570	0.1610*	0.802 (10)
H14C	0.80510	0.37580	0.39200	0.1610*	0.802 (10)
H15A	0.83230	0.35700	0.26410	0.0820*	
H5B1	0.78610	0.05950	0.50330	0.1350*	0.159 (10)
H5B2	0.68140	0.01670	0.51520	0.1350*	0.159 (10)
H6B1	0.88970	−0.00250	0.49740	0.2170*	0.159 (10)
H6B2	0.86300	0.01930	0.45280	0.2170*	0.159 (10)
H6B3	0.76310	−0.02450	0.46860	0.2170*	0.159 (10)
H13C	1.02560	0.38560	0.43760	0.2080*	0.198 (10)
H13D	0.95750	0.38580	0.39290	0.2080*	0.198 (10)
H14D	0.78010	0.34120	0.45120	0.0930*	0.198 (10)
H14E	0.73190	0.37610	0.41470	0.0930*	0.198 (10)
H14F	0.79960	0.39810	0.45580	0.0930*	0.198 (10)
H31A	0.36090	0.00410	0.39790	0.0920*	0.457 (13)
H31B	0.19940	0.02850	0.39920	0.0920*	0.457 (13)
H32A	0.09080	−0.05150	0.39030	0.1550*	0.457 (13)
H32B	0.17820	−0.04620	0.43260	0.1550*	0.457 (13)
H32C	0.25440	−0.07360	0.39510	0.1550*	0.457 (13)

### Atomic displacement parameters (Å^2^)

**Table:**

	*U*^11^	*U*^22^	*U*^33^	*U*^12^	*U*^13^	*U*^23^
Zn1	0.0468 (2)	0.0382 (2)	0.0354 (2)	−0.0078 (2)	−0.0025 (1)	0.0019 (1)
S1	0.0823 (6)	0.0590 (5)	0.0437 (4)	−0.0114 (5)	0.0107 (4)	0.0101 (4)
S2	0.0815 (7)	0.0699 (6)	0.0356 (4)	−0.0123 (5)	−0.0052 (4)	0.0005 (4)
S3	0.0621 (5)	0.0587 (5)	0.0586 (5)	−0.0113 (4)	−0.0147 (4)	−0.0084 (4)
S4	0.0507 (5)	0.0533 (5)	0.0696 (5)	−0.0140 (4)	−0.0031 (4)	0.0120 (4)
S5	0.0476 (4)	0.0468 (4)	0.0379 (3)	−0.0042 (3)	0.0024 (3)	0.0048 (3)
S6	0.0644 (5)	0.0564 (5)	0.0416 (4)	−0.0071 (4)	−0.0033 (4)	−0.0026 (3)
S7	0.0627 (5)	0.0569 (5)	0.0542 (5)	−0.0169 (4)	−0.0044 (4)	−0.0068 (4)
S8B	0.115 (4)	0.0484 (19)	0.0562 (13)	−0.032 (2)	0.008 (2)	−0.0002 (13)
N1	0.0487 (15)	0.0380 (13)	0.0371 (12)	−0.0066 (11)	−0.0021 (11)	0.0024 (10)
N2	0.0461 (14)	0.0394 (13)	0.0375 (12)	−0.0025 (11)	−0.0038 (11)	−0.0004 (10)
N3	0.0404 (14)	0.0378 (12)	0.0385 (12)	−0.0053 (10)	−0.0021 (10)	0.0020 (10)
N4	0.0402 (13)	0.0365 (12)	0.0389 (12)	−0.0016 (10)	0.0015 (10)	0.0020 (9)
N5	0.0409 (13)	0.0369 (12)	0.0365 (12)	−0.0035 (10)	0.0002 (10)	0.0004 (9)
N6	0.0431 (13)	0.0378 (12)	0.0390 (13)	−0.0019 (11)	−0.0014 (10)	−0.0007 (10)
N7	0.0483 (14)	0.0354 (12)	0.0380 (12)	−0.0060 (11)	0.0005 (11)	0.0008 (10)
N8	0.0494 (15)	0.0392 (13)	0.0414 (13)	−0.0053 (11)	0.0014 (11)	0.0021 (10)
C1	0.0440 (17)	0.0424 (16)	0.0389 (14)	0.0007 (13)	0.0022 (12)	0.0033 (12)
C2	0.0455 (17)	0.0425 (16)	0.0382 (14)	0.0020 (13)	−0.0013 (12)	−0.0011 (12)
C3	0.0503 (18)	0.0477 (16)	0.0359 (15)	0.0008 (14)	0.0029 (13)	−0.0002 (12)
C4	0.0557 (18)	0.0447 (16)	0.0349 (14)	−0.0032 (14)	0.0040 (14)	0.0034 (12)
C7	0.123 (4)	0.082 (3)	0.060 (2)	−0.027 (3)	−0.024 (2)	−0.004 (2)
C8	0.105 (4)	0.133 (4)	0.066 (3)	−0.039 (3)	−0.008 (3)	−0.020 (3)
C9	0.0412 (16)	0.0390 (15)	0.0418 (15)	−0.0005 (12)	−0.0030 (12)	−0.0025 (12)
C10	0.0366 (15)	0.0373 (14)	0.0429 (15)	0.0003 (12)	−0.0003 (12)	0.0022 (12)
C11	0.0407 (16)	0.0375 (14)	0.0491 (17)	−0.0036 (13)	−0.0026 (13)	0.0031 (13)
C12	0.0434 (17)	0.0410 (16)	0.0471 (16)	−0.0044 (13)	−0.0080 (14)	−0.0040 (12)
C15	0.072 (2)	0.052 (2)	0.083 (3)	0.0030 (18)	0.005 (2)	0.0121 (18)
C16	0.106 (4)	0.060 (2)	0.109 (4)	0.005 (2)	0.012 (3)	0.026 (2)
C17	0.0372 (15)	0.0372 (14)	0.0373 (14)	0.0019 (12)	0.0020 (12)	0.0051 (11)
C18	0.0392 (16)	0.0392 (14)	0.0376 (14)	0.0037 (12)	−0.0016 (12)	0.0006 (12)
C19	0.0423 (16)	0.0403 (15)	0.0325 (13)	0.0042 (12)	−0.0015 (11)	0.0013 (11)
C20	0.0395 (15)	0.0390 (15)	0.0371 (14)	0.0010 (12)	0.0007 (12)	0.0033 (11)
C21	0.063 (2)	0.0427 (17)	0.060 (2)	−0.0037 (16)	0.0114 (17)	−0.0012 (14)
C22	0.098 (3)	0.055 (2)	0.073 (3)	−0.013 (2)	0.014 (2)	0.0118 (18)
C23	0.062 (2)	0.067 (2)	0.0428 (17)	0.0045 (17)	0.0014 (15)	−0.0003 (15)
C24	0.088 (3)	0.105 (3)	0.052 (2)	0.001 (3)	−0.010 (2)	−0.017 (2)
C25	0.0410 (16)	0.0350 (14)	0.0423 (15)	0.0007 (12)	−0.0010 (12)	−0.0026 (12)
C26	0.0427 (16)	0.0365 (15)	0.0418 (15)	−0.0008 (13)	0.0006 (13)	0.0010 (12)
C27B	0.034 (6)	0.033 (6)	0.057 (7)	−0.004 (4)	−0.010 (4)	−0.016 (5)
C28	0.0418 (16)	0.0386 (15)	0.0445 (17)	−0.0021 (13)	−0.0020 (13)	−0.0014 (12)
C29	0.091 (3)	0.101 (3)	0.059 (2)	−0.031 (3)	0.007 (2)	−0.021 (2)
C30	0.100 (3)	0.117 (4)	0.074 (3)	−0.020 (3)	−0.006 (3)	−0.033 (3)
S8A	0.063 (2)	0.058 (3)	0.072 (2)	−0.0233 (17)	−0.0153 (19)	0.021 (2)
C27A	0.043 (9)	0.048 (8)	0.040 (7)	−0.017 (5)	0.002 (6)	0.021 (6)

### Geometric parameters (Å, °)

**Table:**

Zn1—N1	2.004 (2)	C27A—C28	1.415 (14)
Zn1—N3	1.994 (2)	C27B—C28	1.350 (13)
Zn1—N5	2.004 (2)	C29—C30	1.507 (7)
Zn1—N7	1.994 (2)	C31A—C32A	1.531 (18)
Zn1—S5^i^	2.6364 (9)	C31B—C32B	1.492 (14)
S1—C4	1.747 (3)	C5A—H5A1	0.9700
S1—C5A	1.801 (5)	C5A—H5A2	0.9700
S1—C5B	1.75 (3)	C5B—H5B1	0.9700
S2—C3	1.738 (3)	C5B—H5B2	0.9700
S2—C7	1.787 (5)	C6A—H6A3	0.9600
S3—C12	1.752 (3)	C6A—H6A1	0.9600
S3—C13A	1.817 (5)	C6A—H6A2	0.9600
S3—C13B	1.75 (3)	C6B—H6B2	0.9600
S4—C11	1.748 (3)	C6B—H6B1	0.9600
S4—C15	1.820 (4)	C6B—H6B3	0.9600
S5—C20	1.750 (3)	C7—H7B	0.9700
S5—C21	1.820 (3)	C7—H7A	0.9700
S6—C19	1.734 (3)	C8—H8B	0.9600
S6—C23	1.796 (3)	C8—H8A	0.9600
S7—C28	1.728 (3)	C8—H8C	0.9600
S7—C29	1.780 (4)	C13A—H13B	0.9700
S8A—C27A	1.81 (2)	C13A—H13A	0.9700
S8A—C31A	1.617 (12)	C13B—H13C	0.9700
S8B—C27B	1.713 (16)	C13B—H13D	0.9700
S8B—C31B	1.625 (9)	C14A—H14C	0.9600
N1—C2	1.365 (4)	C14A—H14A	0.9600
N1—C1	1.362 (4)	C14A—H14B	0.9600
N2—C2	1.327 (4)	C14B—H14E	0.9600
N2—C9	1.339 (4)	C14B—H14D	0.9600
N3—C10	1.356 (4)	C14B—H14F	0.9600
N3—C9	1.359 (4)	C15—H15A	0.9700
N4—C17	1.325 (4)	C15—H15B	0.9700
N4—C10	1.342 (4)	C16—H16B	0.9600
N5—C17	1.368 (4)	C16—H16C	0.9600
N5—C18	1.350 (3)	C16—H16A	0.9600
N6—C25	1.329 (4)	C21—H21B	0.9700
N6—C18	1.334 (4)	C21—H21A	0.9700
N7—C25	1.364 (4)	C22—H22A	0.9600
N7—C26	1.369 (4)	C22—H22B	0.9600
N8—C1	1.338 (4)	C22—H22C	0.9600
N8—C26	1.328 (4)	C23—H23B	0.9700
C1—C4	1.452 (4)	C23—H23A	0.9700
C2—C3	1.471 (4)	C24—H24B	0.9600
C3—C4	1.365 (4)	C24—H24A	0.9600
C5A—C6A	1.486 (9)	C24—H24C	0.9600
C5B—C6B	1.25 (5)	C29—H29B	0.9700
C7—C8	1.485 (6)	C29—H29A	0.9700
C9—C12	1.458 (4)	C30—H30C	0.9600
C10—C11	1.455 (4)	C30—H30A	0.9600
C11—C12	1.359 (4)	C30—H30B	0.9600
C13A—C14A	1.548 (10)	C31A—H31B	0.9700
C13B—C14B	1.49 (3)	C31A—H31A	0.9700
C15—C16	1.493 (6)	C31B—H31D	0.9700
C17—C20	1.466 (4)	C31B—H31C	0.9700
C18—C19	1.472 (4)	C32A—H32B	0.9600
C19—C20	1.371 (4)	C32A—H32C	0.9600
C21—C22	1.505 (5)	C32A—H32A	0.9600
C23—C24	1.534 (5)	C32B—H32D	0.9600
C25—C28	1.468 (4)	C32B—H32E	0.9600
C26—C27B	1.542 (19)	C32B—H32F	0.9600
C26—C27A	1.37 (2)		
			
Zn1···H23B^i^	3.5500	C21···N4	2.978 (4)
S1···S2	3.3706 (13)	C21···S4^i^	3.660 (4)
S1···N8	3.207 (2)	C23···S5	3.221 (4)
S2···C5B	3.57 (3)	C25···S7^iv^	3.440 (3)
S2···S3^ii^	3.7036 (13)	C25···C18^i^	3.598 (4)
S2···N2	3.353 (2)	C25···C19^i^	3.535 (4)
S2···S1	3.3706 (13)	C26···S6^i^	3.569 (3)
S3···S4	3.5017 (14)	C27B···S6^i^	3.66 (2)
S3···N2	3.225 (3)	C28···S7^iv^	3.655 (3)
S3···S2^iii^	3.7036 (13)	C29···S8A^iv^	3.621 (8)
S4···C21^iv^	3.660 (4)	C29···N6	3.026 (5)
S4···S3	3.5017 (14)	C31A···N8	3.129 (12)
S4···N4	3.221 (3)	C31B···N8	3.089 (9)
S5···C23	3.221 (4)	C32B···C13B^vi^	3.30 (3)
S5···N4	3.268 (2)	C32B···C5B^v^	3.42 (3)
S5···S6	3.6711 (12)	C1···H23B^i^	2.8900
S6···S5	3.6711 (12)	C2···H8B^ii^	2.9900
S6···C27B^iv^	3.66 (2)	C2···H7A	3.0800
S6···N6	2.976 (2)	C3···H24C^iv^	3.1000
S6···C26^iv^	3.569 (3)	C3···H8B^ii^	2.9800
S6···C9^i^	3.560 (3)	C4···H6A3	2.7700
S7···C25^i^	3.440 (3)	C5B···H32E^v^	2.7700
S7···S8A	3.282 (7)	C6A···H30B^iv^	3.0600
S7···S8B	3.296 (5)	C6B···H6B1^viii^	2.7300
S7···N6	3.311 (3)	C7···H13A	3.0500
S7···C28^i^	3.655 (3)	C9···H23A^iv^	3.1000
S8A···S7	3.282 (7)	C9···H13A	3.0200
S8A···N8	3.328 (8)	C9···H22A^i^	2.8900
S8A···C29^i^	3.621 (8)	C10···H15A	2.9500
S8B···S7	3.296 (5)	C12···H14C	2.8700
S8B···N8	3.318 (7)	C13B···H32E^ix^	2.7000
S1···H13B^ii^	3.1100	C14A···H6B3^ix^	3.0800
S1···H5A1^v^	2.8600	C21···H16C	3.0800
S1···H5B2^v^	3.1800	C22···H14E^iv^	3.0400
S1···H13C^ii^	3.0500	C26···H31D	3.0400
S2···H5B1	2.8200	C32B···H5B2^v^	2.8700
S2···H24C^iv^	3.1800	C32B···H13C^vi^	2.7300
S3···H23A^iv^	3.1200	C32B···H5B1^v^	3.0000
S3···H8A^iii^	3.1600	C32B···H13D^vi^	3.0100
S4···H21A^iv^	2.7700	H5A1···S1^v^	2.8600
S5···H23A	3.1200	H5A2···N8	2.9000
S5···H23B	2.6900	H6A3···C4	2.7700
S6···H29A	3.1300	H5B1···C32B^v^	3.0000
S7···H16A^vi^	3.1800	H5B1···S2	2.8200
S7···H16B^vii^	3.0700	H5B1···H32E^v^	2.3800
S8A···H29B^i^	2.7700	H5B2···C32B^v^	2.8700
S8B···H16A^vi^	3.0600	H5B2···H32E^v^	2.3200
N1···N3	2.772 (3)	H5B2···S1^v^	3.1800
N1···N7	2.786 (3)	H7A···N2	2.5000
N1···C9	2.885 (4)	H7A···C2	3.0800
N1···C26	2.908 (4)	H7B···H13A	2.5000
N2···S3	3.225 (3)	H6B1···H6B1^viii^	1.9500
N2···C7	3.014 (5)	H6B1···C6B^viii^	2.7300
N2···S2	3.353 (2)	H8A···S3^ii^	3.1600
N2···C13A	3.420 (5)	H8B···C3^iii^	2.9800
N3···N5	2.779 (3)	H8B···C2^iii^	2.9900
N3···C2	2.896 (4)	H6B3···H14B^vi^	2.4600
N3···C17	2.890 (3)	H6B3···C14A^vi^	3.0800
N3···N1	2.772 (3)	H13A···C7	3.0500
N4···S4	3.221 (3)	H13A···N2	2.7700
N4···S5	3.268 (2)	H13A···C9	3.0200
N4···C21	2.978 (4)	H13A···H7B	2.5000
N4···C18^iv^	3.376 (4)	H13B···S1^iii^	3.1100
N4···C15	3.374 (4)	H13C···S1^iii^	3.0500
N5···C25	2.901 (4)	H13C···C32B^ix^	2.7300
N5···N3	2.779 (3)	H13C···H32E^ix^	1.9500
N5···C10	2.893 (4)	H13D···C32B^ix^	3.0100
N5···N7	2.787 (3)	H13D···H31C^ix^	2.5700
N6···S7	3.311 (3)	H14B···H6B3^ix^	2.4600
N6···S6	2.976 (2)	H14C···C12	2.8700
N6···C29	3.026 (5)	H14E···C22^i^	3.0400
N7···C18	2.879 (3)	H15A···C10	2.9500
N7···N5	2.787 (3)	H15A···N4	2.7100
N7···C1	2.892 (4)	H15A···H21A	2.4600
N7···N1	2.786 (3)	H16A···S8B^ix^	3.0600
N7···C20^i^	3.444 (4)	H16A···S7^ix^	3.1800
N8···S8B	3.318 (7)	H16B···S7^x^	3.0700
N8···S8A	3.328 (8)	H16C···C21	3.0800
N8···S1	3.207 (2)	H16C···H22B	2.4800
N8···C31B	3.089 (9)	H21A···N4	2.7800
N8···C31A	3.129 (12)	H21A···H15A	2.4600
N2···H13A	2.7700	H21A···S4^i^	2.7700
N2···H7A	2.5000	H21B···N4	2.6600
N4···H15A	2.7100	H22A···C9^iv^	2.8900
N4···H21B	2.6600	H22B···H16C	2.4800
N4···H21A	2.7800	H23A···S3^i^	3.1200
N6···H29B	2.8400	H23A···C9^i^	3.1000
N6···H29A	2.6700	H23A···S5	3.1200
N8···H31D	2.5300	H23B···Zn1^iv^	3.5500
N8···H5A2	2.9000	H23B···C1^iv^	2.8900
N8···H31A	2.6000	H23B···S5	2.6900
C5B···C32B^v^	3.42 (3)	H24A···H31B^iv^	2.3100
C5B···S2	3.57 (3)	H24C···S2^i^	3.1800
C6B···C6B^viii^	3.59 (5)	H24C···C3^i^	3.1000
C7···N2	3.014 (5)	H29A···S6	3.1300
C9···S6^iv^	3.560 (3)	H29A···N6	2.6700
C10···C18^iv^	3.567 (4)	H29B···N6	2.8400
C10···C19^iv^	3.471 (4)	H29B···S8A^iv^	2.7700
C11···C20^iv^	3.567 (4)	H30B···C6A^i^	3.0600
C13A···N2	3.420 (5)	H31A···N8	2.6000
C13B···C32B^ix^	3.30 (3)	H31B···H24A^i^	2.3100
C15···N4	3.374 (4)	H31C···H13D^vi^	2.5700
C17···C18^iv^	3.485 (4)	H31D···N8	2.5300
C18···C17^i^	3.485 (4)	H31D···C26	3.0400
C18···C25^iv^	3.598 (4)	H32E···H5B1^v^	2.3800
C18···C10^i^	3.567 (4)	H32E···H5B2^v^	2.3200
C18···N4^i^	3.376 (4)	H32E···C13B^vi^	2.7000
C19···C25^iv^	3.535 (4)	H32E···H13C^vi^	1.9500
C19···C10^i^	3.471 (4)	H32E···C5B^v^	2.7700
C20···C11^i^	3.567 (4)		
			
N1—Zn1—N3	87.77 (10)	S1—C5B—H5B2	102.00
N1—Zn1—N5	158.84 (10)	S1—C5B—H5B1	102.00
N1—Zn1—N7	88.35 (9)	H6A1—C6A—H6A3	109.00
S5^i^—Zn1—N1	99.45 (7)	H6A1—C6A—H6A2	110.00
N3—Zn1—N5	88.07 (10)	C5A—C6A—H6A1	109.00
N3—Zn1—N7	159.68 (10)	C5A—C6A—H6A2	109.00
S5^i^—Zn1—N3	99.71 (7)	C5A—C6A—H6A3	109.00
N5—Zn1—N7	88.40 (9)	H6A2—C6A—H6A3	109.00
S5^i^—Zn1—N5	101.71 (7)	C5B—C6B—H6B2	109.00
S5^i^—Zn1—N7	100.60 (8)	C5B—C6B—H6B3	109.00
C4—S1—C5A	101.1 (2)	C5B—C6B—H6B1	109.00
C4—S1—C5B	107.8 (9)	H6B1—C6B—H6B3	110.00
C3—S2—C7	107.19 (17)	H6B2—C6B—H6B3	109.00
C12—S3—C13A	101.67 (19)	H6B1—C6B—H6B2	110.00
C12—S3—C13B	105.0 (12)	C8—C7—H7A	110.00
C11—S4—C15	101.02 (15)	H7A—C7—H7B	108.00
C20—S5—C21	107.54 (15)	S2—C7—H7B	110.00
Zn1^iv^—S5—C20	98.74 (9)	C8—C7—H7B	110.00
Zn1^iv^—S5—C21	105.22 (11)	S2—C7—H7A	110.00
C19—S6—C23	107.52 (15)	C7—C8—H8A	109.00
C28—S7—C29	106.76 (17)	H8B—C8—H8C	110.00
C27A—S8A—C31A	112.2 (7)	C7—C8—H8B	109.00
C27B—S8B—C31B	116.0 (7)	C7—C8—H8C	109.00
Zn1—N1—C2	125.99 (18)	H8A—C8—H8B	110.00
C1—N1—C2	108.2 (2)	H8A—C8—H8C	109.00
Zn1—N1—C1	125.59 (19)	S3—C13A—H13B	110.00
C2—N2—C9	123.3 (2)	C14A—C13A—H13A	110.00
Zn1—N3—C9	125.48 (19)	S3—C13A—H13A	110.00
Zn1—N3—C10	126.03 (19)	H13A—C13A—H13B	108.00
C9—N3—C10	107.8 (2)	C14A—C13A—H13B	110.00
C10—N4—C17	123.4 (2)	S3—C13B—H13D	106.00
Zn1—N5—C17	126.32 (18)	C14B—C13B—H13C	106.00
Zn1—N5—C18	125.35 (19)	H13C—C13B—H13D	107.00
C17—N5—C18	108.0 (2)	C14B—C13B—H13D	106.00
C18—N6—C25	122.9 (2)	S3—C13B—H13C	106.00
Zn1—N7—C25	125.69 (18)	H14A—C14A—H14C	109.00
C25—N7—C26	107.8 (2)	C13A—C14A—H14A	110.00
Zn1—N7—C26	126.23 (19)	C13A—C14A—H14B	109.00
C1—N8—C26	123.6 (2)	C13A—C14A—H14C	110.00
N1—C1—C4	109.4 (2)	H14A—C14A—H14B	109.00
N8—C1—C4	122.9 (3)	H14B—C14A—H14C	110.00
N1—C1—N8	127.7 (3)	C13B—C14B—H14D	109.00
N1—C2—N2	127.0 (3)	C13B—C14B—H14E	110.00
N2—C2—C3	124.1 (3)	C13B—C14B—H14F	110.00
N1—C2—C3	108.8 (2)	H14D—C14B—H14E	109.00
S2—C3—C2	130.6 (2)	H14D—C14B—H14F	109.00
C2—C3—C4	106.4 (2)	H14E—C14B—H14F	110.00
S2—C3—C4	123.0 (2)	S4—C15—H15B	110.00
S1—C4—C1	125.5 (2)	C16—C15—H15A	110.00
S1—C4—C3	127.3 (2)	S4—C15—H15A	110.00
C1—C4—C3	107.2 (2)	H15A—C15—H15B	108.00
S1—C5A—C6A	113.7 (4)	C16—C15—H15B	110.00
S1—C5B—C6B	140 (3)	C15—C16—H16B	109.00
S2—C7—C8	110.2 (3)	C15—C16—H16A	109.00
N3—C9—C12	109.4 (2)	C15—C16—H16C	109.00
N2—C9—C12	123.0 (3)	H16A—C16—H16B	109.00
N2—C9—N3	127.6 (2)	H16A—C16—H16C	109.00
N3—C10—C11	109.4 (2)	H16B—C16—H16C	110.00
N3—C10—N4	127.2 (2)	H21A—C21—H21B	108.00
N4—C10—C11	123.4 (2)	C22—C21—H21A	110.00
S4—C11—C12	127.8 (2)	C22—C21—H21B	110.00
S4—C11—C10	125.4 (2)	S5—C21—H21A	110.00
C10—C11—C12	106.8 (2)	S5—C21—H21B	110.00
S3—C12—C11	127.6 (2)	C21—C22—H22A	109.00
C9—C12—C11	106.5 (2)	C21—C22—H22B	110.00
S3—C12—C9	125.7 (2)	C21—C22—H22C	109.00
S3—C13A—C14A	109.8 (4)	H22A—C22—H22B	109.00
S3—C13B—C14B	124.5 (18)	H22A—C22—H22C	110.00
S4—C15—C16	110.2 (3)	H22B—C22—H22C	109.00
N4—C17—N5	127.1 (2)	S6—C23—H23A	110.00
N5—C17—C20	109.4 (2)	S6—C23—H23B	110.00
N4—C17—C20	123.5 (2)	C24—C23—H23B	110.00
N6—C18—C19	121.7 (2)	H23A—C23—H23B	108.00
N5—C18—N6	128.4 (2)	C24—C23—H23A	110.00
N5—C18—C19	109.9 (2)	H24A—C24—H24B	110.00
S6—C19—C18	116.5 (2)	H24A—C24—H24C	109.00
S6—C19—C20	137.1 (2)	C23—C24—H24A	109.00
C18—C19—C20	106.2 (2)	C23—C24—H24B	109.00
S5—C20—C17	127.0 (2)	C23—C24—H24C	109.00
S5—C20—C19	125.7 (2)	H24B—C24—H24C	110.00
C17—C20—C19	106.5 (2)	S7—C29—H29B	110.00
S5—C21—C22	107.5 (2)	S7—C29—H29A	110.00
S6—C23—C24	107.9 (3)	H29A—C29—H29B	108.00
N6—C25—N7	127.2 (2)	C30—C29—H29A	110.00
N6—C25—C28	123.1 (3)	C30—C29—H29B	110.00
N7—C25—C28	109.6 (2)	C29—C30—H30A	109.00
N8—C26—C27B	123.8 (5)	C29—C30—H30C	109.00
N8—C26—C27A	122.9 (7)	H30A—C30—H30B	109.00
N7—C26—N8	127.0 (2)	C29—C30—H30B	109.00
N7—C26—C27B	109.3 (5)	H30B—C30—H30C	109.00
N7—C26—C27A	109.9 (7)	H30A—C30—H30C	109.00
S8A—C27A—C28	115.9 (14)	S8A—C31A—H31B	109.00
S8A—C27A—C26	131.5 (10)	S8A—C31A—H31A	109.00
C26—C27A—C28	109.9 (13)	C32A—C31A—H31B	109.00
C26—C27B—C28	103.6 (11)	H31A—C31A—H31B	108.00
S8B—C27B—C28	126.7 (10)	C32A—C31A—H31A	109.00
S8B—C27B—C26	126.2 (10)	S8B—C31B—H31C	108.00
S7—C28—C27A	127.5 (9)	S8B—C31B—H31D	108.00
S7—C28—C25	129.8 (2)	H31C—C31B—H31D	107.00
S7—C28—C27B	120.5 (8)	C32B—C31B—H31C	108.00
C25—C28—C27B	109.5 (8)	C32B—C31B—H31D	108.00
C25—C28—C27A	102.7 (9)	C31A—C32A—H32A	109.00
S7—C29—C30	108.9 (3)	C31A—C32A—H32B	109.00
S8A—C31A—C32A	111.3 (9)	C31A—C32A—H32C	109.00
S8B—C31B—C32B	115.6 (7)	H32A—C32A—H32B	110.00
S1—C5A—H5A1	109.00	H32B—C32A—H32C	110.00
S1—C5A—H5A2	109.00	H32A—C32A—H32C	109.00
C6A—C5A—H5A1	109.00	C31B—C32B—H32E	109.00
C6A—C5A—H5A2	109.00	C31B—C32B—H32F	109.00
H5A1—C5A—H5A2	108.00	H32D—C32B—H32F	109.00
C6B—C5B—H5B1	102.00	H32E—C32B—H32F	109.00
C6B—C5B—H5B2	102.00	H32D—C32B—H32E	109.00
H5B1—C5B—H5B2	105.00	C31B—C32B—H32D	110.00
			
N1^iv^—Zn1^iv^—S5—C20	123.48 (11)	C9—N3—C10—N4	173.3 (3)
N3^iv^—Zn1^iv^—S5—C20	−147.19 (11)	Zn1—N3—C10—C11	167.87 (18)
N5^iv^—Zn1^iv^—S5—C20	−57.16 (12)	Zn1—N3—C10—N4	−15.6 (4)
N7^iv^—Zn1^iv^—S5—C20	33.40 (11)	Zn1—N3—C9—N2	13.3 (4)
N1^iv^—Zn1^iv^—S5—C21	−125.55 (14)	C9—N3—C10—C11	−3.2 (3)
N3^iv^—Zn1^iv^—S5—C21	−36.22 (13)	C10—N4—C17—N5	8.1 (5)
N5^iv^—Zn1^iv^—S5—C21	53.81 (14)	C17—N4—C10—N3	2.8 (5)
N7^iv^—Zn1^iv^—S5—C21	144.37 (13)	C17—N4—C10—C11	178.9 (3)
N3—Zn1—N1—C1	−170.8 (2)	C10—N4—C17—C20	−171.0 (3)
N5—Zn1—N1—C1	−92.0 (4)	C17—N5—C18—N6	176.0 (3)
N7—Zn1—N1—C1	−10.8 (2)	Zn1—N5—C17—C20	174.37 (18)
S5^i^—Zn1—N1—C1	89.7 (2)	Zn1—N5—C17—N4	−4.8 (4)
N3—Zn1—N1—C2	15.2 (2)	Zn1—N5—C18—N6	2.4 (4)
N5—Zn1—N1—C2	94.0 (3)	C18—N5—C17—C20	0.9 (3)
N7—Zn1—N1—C2	175.3 (2)	C18—N5—C17—N4	−178.3 (3)
S5^i^—Zn1—N1—C2	−84.2 (2)	C17—N5—C18—C19	−1.1 (3)
N1—Zn1—N3—C9	−17.4 (2)	Zn1—N5—C18—C19	−174.63 (18)
N5—Zn1—N3—C9	−176.7 (2)	C18—N6—C25—C28	−172.6 (3)
N7—Zn1—N3—C9	−96.6 (3)	C18—N6—C25—N7	2.0 (5)
S5^i^—Zn1—N3—C9	81.8 (2)	C25—N6—C18—C19	166.6 (3)
N1—Zn1—N3—C10	173.0 (2)	C25—N6—C18—N5	−10.2 (5)
N5—Zn1—N3—C10	13.7 (2)	C26—N7—C25—N6	−172.9 (3)
N7—Zn1—N3—C10	93.9 (3)	Zn1—N7—C25—N6	12.7 (4)
S5^i^—Zn1—N3—C10	−87.8 (2)	Zn1—N7—C26—N8	−10.3 (4)
N1—Zn1—N5—C17	−83.4 (3)	C25—N7—C26—C27B	−4.2 (9)
N3—Zn1—N5—C17	−4.6 (2)	Zn1—N7—C25—C28	−172.11 (19)
N7—Zn1—N5—C17	−164.6 (2)	Zn1—N7—C26—C27B	170.3 (9)
S5^i^—Zn1—N5—C17	94.9 (2)	C25—N7—C26—N8	175.3 (3)
N1—Zn1—N5—C18	89.1 (4)	C26—N7—C25—C28	2.3 (3)
N3—Zn1—N5—C18	167.8 (2)	C26—N8—C1—C4	−177.1 (3)
N7—Zn1—N5—C18	7.8 (2)	C1—N8—C26—N7	−0.1 (5)
S5^i^—Zn1—N5—C18	−92.7 (2)	C26—N8—C1—N1	2.7 (5)
N1—Zn1—N7—C25	−173.6 (2)	C1—N8—C26—C27B	179.2 (11)
N3—Zn1—N7—C25	−94.6 (3)	N1—C1—C4—C3	0.3 (3)
N5—Zn1—N7—C25	−14.5 (2)	N8—C1—C4—S1	−1.1 (4)
S5^i^—Zn1—N7—C25	87.1 (2)	N1—C1—C4—S1	179.1 (2)
N1—Zn1—N7—C26	13.0 (2)	N8—C1—C4—C3	−179.9 (3)
N3—Zn1—N7—C26	92.0 (3)	N1—C2—C3—C4	0.6 (3)
N5—Zn1—N7—C26	172.1 (2)	N1—C2—C3—S2	177.3 (2)
S5^i^—Zn1—N7—C26	−86.3 (2)	N2—C2—C3—C4	−177.0 (3)
C4—S1—C5A—C6A	62.8 (5)	N2—C2—C3—S2	−0.3 (4)
C5A—S1—C4—C1	72.1 (3)	C2—C3—C4—S1	−179.4 (2)
C5A—S1—C4—C3	−109.2 (3)	S2—C3—C4—S1	3.6 (4)
C3—S2—C7—C8	−178.1 (3)	S2—C3—C4—C1	−177.5 (2)
C7—S2—C3—C4	−166.6 (3)	C2—C3—C4—C1	−0.5 (3)
C7—S2—C3—C2	17.2 (3)	N2—C9—C12—C11	177.1 (3)
C13A—S3—C12—C11	119.6 (3)	N3—C9—C12—S3	−176.2 (2)
C12—S3—C13A—C14A	−74.0 (4)	N2—C9—C12—S3	2.4 (4)
C13A—S3—C12—C9	−66.8 (3)	N3—C9—C12—C11	−1.4 (3)
C15—S4—C11—C10	62.0 (3)	N3—C10—C11—S4	−178.3 (2)
C11—S4—C15—C16	−169.2 (3)	N4—C10—C11—C12	−174.3 (3)
C15—S4—C11—C12	−118.8 (3)	N3—C10—C11—C12	2.4 (3)
Zn1^iv^—S5—C20—C19	−89.6 (2)	N4—C10—C11—S4	5.0 (4)
C21—S5—C20—C19	161.3 (3)	C10—C11—C12—C9	−0.5 (3)
Zn1^iv^—S5—C20—C17	79.2 (2)	C10—C11—C12—S3	174.1 (2)
C20—S5—C21—C22	−162.9 (2)	S4—C11—C12—S3	−5.2 (4)
C21—S5—C20—C17	−29.9 (3)	S4—C11—C12—C9	−179.8 (2)
Zn1^iv^—S5—C21—C22	92.6 (2)	N5—C17—C20—C19	−0.4 (3)
C23—S6—C19—C20	−6.1 (4)	N5—C17—C20—S5	−170.9 (2)
C23—S6—C19—C18	−179.9 (2)	N4—C17—C20—S5	8.3 (4)
C19—S6—C23—C24	172.6 (2)	N4—C17—C20—C19	178.9 (3)
C28—S7—C29—C30	164.8 (3)	N6—C18—C19—C20	−176.5 (3)
C29—S7—C28—C27B	−154.9 (11)	N5—C18—C19—C20	0.8 (3)
C29—S7—C28—C25	30.2 (3)	N5—C18—C19—S6	176.49 (19)
C27B—S8B—C31B—C32B	158.9 (12)	N6—C18—C19—S6	−0.8 (4)
C31B—S8B—C27B—C26	11 (2)	C18—C19—C20—C17	−0.3 (3)
C31B—S8B—C27B—C28	166.6 (17)	S6—C19—C20—S5	−3.8 (5)
C2—N1—C1—C4	0.2 (3)	C18—C19—C20—S5	170.4 (2)
Zn1—N1—C2—N2	−8.1 (4)	S6—C19—C20—C17	−174.6 (2)
Zn1—N1—C1—N8	5.5 (4)	N7—C25—C28—C27B	0.7 (11)
Zn1—N1—C1—C4	−174.7 (2)	N7—C25—C28—S7	176.0 (2)
C2—N1—C1—N8	−179.6 (3)	N6—C25—C28—C27B	176.1 (11)
Zn1—N1—C2—C3	174.35 (19)	N6—C25—C28—S7	−8.6 (4)
C1—N1—C2—N2	177.1 (3)	N7—C26—C27B—C28	4.5 (15)
C1—N1—C2—C3	−0.5 (3)	N8—C26—C27B—C28	−175.0 (7)
C2—N2—C9—N3	1.0 (5)	N8—C26—C27B—S8B	−15 (2)
C9—N2—C2—C3	173.4 (3)	N7—C26—C27B—S8B	164.4 (13)
C9—N2—C2—N1	−3.8 (5)	S8B—C27B—C28—C25	−162.7 (16)
C2—N2—C9—C12	−177.3 (3)	C26—C27B—C28—S7	−178.8 (6)
Zn1—N3—C9—C12	−168.27 (18)	C26—C27B—C28—C25	−3.0 (15)
C10—N3—C9—C12	2.9 (3)	S8B—C27B—C28—S7	22 (2)
C10—N3—C9—N2	−175.6 (3)		

Symmetry codes: (i) *x*−1/2, *y*, −*z*+1/2; (ii) *x*−1/2, −*y*+1/2, −*z*+1; (iii) *x*+1/2, −*y*+1/2, −*z*+1; (iv) *x*+1/2, *y*, −*z*+1/2; (v) −*x*+1, −*y*, −*z*+1; (vi) −*x*+3/2, *y*−1/2, *z*; (vii) −*x*+1, *y*−1/2, −*z*+1/2; (viii) −*x*+2, −*y*, −*z*+1; (ix) −*x*+3/2, *y*+1/2, *z*; (x) −*x*+1, *y*+1/2, −*z*+1/2.

### Hydrogen-bond geometry (Å, °)

**Table:**

Cg1, Cg2 and Cg3 are the centroids of the N3/C9–C12, Zn1/N1/N7/N8/C1/C26 and Zn1/N3/N4/N5/C10/C17 rings, respectively.

**Table:**

*D*—H···*A*	*D*—H	H···*A*	*D*···*A*	*D*—H···*A*
C5A—H5A1···S1^v^	0.97	2.86	3.764 (5)	155
C7—H7A···N2	0.97	2.50	3.014 (5)	113
C21—H21A···S4^i^	0.97	2.77	3.660 (4)	154
C23—H23B···S5	0.97	2.69	3.221 (4)	115
C31B—H31D···N8	0.97	2.53	3.089 (9)	116
C21—H21B···Cg3^iv^	0.97	2.86	3.536 (3)	128
C22—H22A···Cg1^iv^	0.96	2.90	3.699 (4)	142
C23—H23B···Cg2^iv^	0.97	2.81	3.641 (4)	144

Symmetry codes: (v) −*x*+1, −*y*, −*z*+1; (i) *x*−1/2, *y*, −*z*+1/2; (iv) *x*+1/2, *y*, −*z*+1/2.
